# Pathways to adaptive functioning in autism from early childhood to adolescence

**DOI:** 10.1002/aur.2785

**Published:** 2022-07-28

**Authors:** Susie Chandler, Virginia Carter Leno, Philippa White, Isabel Yorke, Matthew J. Hollocks, Gillian Baird, Andrew Pickles, Emily Simonoff, Tony Charman

**Affiliations:** ^1^ Institute of Psychiatry, Psychology & Neuroscience King's College London London UK; ^2^ South London and Maudsley NHS Foundation Trust (SLaM) London UK; ^3^ Guy's and St Thomas' NHS Foundation Trust London UK; ^4^ Maudsley Biomedical Research Centre for Mental Health London UK

**Keywords:** adaptive function, ADHD, autism, behavioral problems, emotional problems

## Abstract

Adaptive functioning is lower in many autistic individuals to a greater extent than would be expected based on IQ. However, the clinical features associated with these difficulties are less well understood. This study examines longitudinal and contemporaneous associations of adaptive functioning in autistic youth across a wide ability range. Parent‐reported autism symptoms, co‐occurring emotional, behavioral and attention deficit hyperactivity disorder (ADHD) symptoms, and IQ were assessed in early childhood (*M* age 7 years; T1) and 6 years later in adolescence (*M* age 13 years; T2) in 179 autistic youth. Adaptive functioning was assessed at T2. Structural equation modeling estimated pathways to adaptive functioning from autism, and psychiatric symptoms at T1 and T2, testing whether associations were driven by continuity of behaviors from T1 to T2 or their contemporaneous effect at T2, or both, controlling for T1 IQ. Lower adaptive functioning at T2 was associated with higher T1 and T2 ADHD symptoms (*β* = −0.14, and *β* = −0.21) but not behavioral nor emotional symptoms at either timepoint. Lower adaptive functioning at T2 was also associated with lower T1 IQ (*β* = 0.43) and higher social communication symptoms (*β* = −0.37) at T2 but not T1, but the relationship with ADHD symptoms remained. Paths were not moderated by sex or IQ. Increased symptoms of ADHD, both in early childhood and contemporaneously, were associated with reduced adaptive functioning in adolescence. Co‐occurring ADHD may be a modifiable risk factor for adaptive function impairments and should be routinely assessed and when present evidence‐based treatments initiated which may benefit adaptive functioning outcomes.

**Lay Summary:**

Adaptive functioning is lower in many autistic individuals to a greater extent than would be expected based on IQ. However, the clinical features associated with these difficulties are less well understood. In a community sample higher attention deficit/hyperactivity disorder (ADHD) symptoms, but not emotional or behavioral symptoms, in both early childhood and contemporaneously were associated with lower adaptive functioning in autistic adolescents. Co‐occurring ADHD may be a modifiable risk factor for adaptive function difficulties in autism.

## INTRODUCTION

Adaptive functioning describes how well an individual manages the demands of everyday life and can cope independently in areas such as socialization, communication, self‐care, home living, leisure, and community participation. Despite wide heterogeneity, it is well established that many autistic individuals^1^ have difficulties in adaptive functioning. Although adaptive functioning is associated with cognitive ability (IQ), in many autistic individuals adaptive functioning is relatively impaired compared to IQ, particularly in higher‐ability individuals (Alvares et al., 2020; Charman et al., 2011; Chatham et al., 2018; Kanne et al., 2011; Tillmann et al., 2019). The discrepancy between IQ and adaptive functioning increases from early childhood to adulthood (Bal et al., 2015; Tillmann et al., 2019). This suggests that many autistic individuals have difficulty translating their cognitive potential into functional independence. Adaptive functioning is an important determinant of outcome in domains such as educational attainment (Clarke et al., 2020; de Bildt et al., 2005), employment (Taylor & Mailick, 2014) and level of independent living (Farley et al., 2009).

The clinical features associated with difficulties in adaptive functioning in autism are less well understood. Longitudinal studies have shown that the strongest associations of higher adaptive functioning outcome and trajectory in autistic individuals are higher early adaptive functioning, IQ, and language ability (Bal et al., 2015; Clarke et al., 2020; Szatmari et al., 2015, 2021). Several cross‐sectional studies have found that lower adaptive functioning is associated with higher autistic symptom severity (Duncan & Bishop, 2015; Kanne et al., 2011; Tillmann et al., 2019) and higher co‐occurring psychiatric symptoms, including attention deficit/hyperactivity disorder (ADHD) in children (Sikora et al., 2012; Yerys et al., 2019) and ADHD, depression, and anxiety in adults (Kraper et al., 2017). However, neither Tillmann et al. (2019) nor Duncan and Bishop (2015) found that ADHD or internalizing symptoms were associated with adaptive functioning when age, IQ and symptom severity were accounted for.

To date, most studies examining the association between co‐occurring psychiatric symptoms and adaptive functioning have used cross‐sectional designs and focused on higher‐ability individuals. Identifying factors early in development that are associated with adaptive functioning outcomes is potentially important, especially if they may be amenable to modification through evidence‐based interventions. To understand the pathways to adaptive functioning, we examined longitudinal and contemporaneous associations between adaptive functioning, autism symptoms and co‐occurring psychiatric symptoms (emotional, behavioral and ADHD symptoms), whilst accounting for homotypic and heterotypic associations in these symptom domains over time, and variation in IQ. We tested whether autism and co‐occurring psychiatric symptoms in early childhood are associated with later adaptive functioning or whether associations are contemporaneous in adolescence, taking account of the expected continuity within each domain over time. Few previous studies have determined whether associations with adaptive behavior differ by sex and IQ. Therefore, where associations were identified we tested whether these were moderated by IQ or sex.

## METHODS

### Participants

This study included participants from the QUEST study (Salazar et al., 2015), a longitudinal community sample recruited when children were between 4 and 8 years old (Time 1; age *M* (SD) 6.8 (1.2), 4–9 years, *n* = 277) and followed up throughout childhood as part of the IAMHealth project (http://iamhealthkcl.net/). All children with an autism spectrum disorder diagnosis, born between September 2000 and September 2004, living in two health districts (one inner London, one outer London) were invited to participate (*n* = 447). Of that number responses were received from 362 (81%), with 277 parents (62%) completing study questionnaires. Upon entry to the study, participants were stratified into an “intensively studied” (intensive; *n* = 101) and “extensively studied” group (extensive; *n* = 176), which was maintained throughout subsequent waves of data collection. All females were included in the stratified intensive sample, while boys were selected at random whilst stratified on IQ (<70/≥70), age upon entry to the study (<6.7/≥6.8 years) and autism symptom severity (using the Social Communication Questionnaire [SCQ; Rutter, Bailey, et al., 2003] total score, <21/≥22) to ensure equal distribution in the full versus stratified sample. The cohort was followed 6 years later (Time 2; age *M* (SD) 13.5 (1.2), 11–15 years), with the intensive participants and their caregivers completing more in‐depth assessments at each time point. All participants had a clinical diagnosis of autism at recruitment, and the intensive group had their diagnosis confirmed at Time 2 with the Autism Diagnostic Observation Schedule‐2 (ADOS‐2; Lord et al., 2012) and a subset (*n* = 53) also with the Autism Diagnostic Interview‐Revised (ADI‐R; Rutter, Le Couteur, et al., 2003) to which the recommended autism spectrum cut‐off (Risi et al., 2006) was applied. All participants were above the autism spectrum threshold on either or both instruments. This study is based on the 179 participants with data at Time 1 (T1) and Time 2 (T2).

All participating families gave their written informed consent, and the study was approved by Guy's Hospital REC (08/H0804/37) at T1, and Camden and King's Cross Ethics REC (14/LO/2098) at T2.

### Measures

#### Autism symptomatology

The Social Communication Questionnaire (SCQ; Rutter et al., 2003) was completed by parents/carers at T1 (Lifetime version) and T2 (Current version) providing total scores and domain scores for Social Communication and Interaction (SCI) and Restricted and Repetitive Behaviors (RRBs) (Evans et al., 2019).

#### Co‐occurring psychiatric symptoms

At T1, the parent/carer report Developmental Behavior Checklist version (DBC) (Einfeld & Tonge, 2002) was used. It has satisfactory psychometric properties and concurrent validity with other psychopathology measures, particularly in samples of children and adolescents with intellectual disability (Einfeld et al., 2006; Einfeld & Tonge, 1995), and has been used to describe psychopathology in autistic children and adolescents (Brereton et al., 2006).

At T2, the parent/carer report Strengths and Difficulties Questionnaire (SDQ; Goodman et al., 2000) was used. Its psychometric properties are well established within general population samples (Goodman, 2001) and autistic children and adolescents (Findon et al., 2016), and its clinical utility demonstrated in populations with intellectual disability (Murray et al., 2020). See Supplementary Materials for additional details of DBC and SDQ.

For both the DBC and SDQ, a higher score indicates more problems. We focused on psychiatric symptoms across three key domains: ADHD symptoms (DBC Hyperactivity scale at T1, SDQ Hyperactivity‐inattention scale at T2); behavioral symptoms (DBC Disruptive/Anti‐social scale at T1, SDQ Conduct Problems at T2); and emotional symptoms (DBC Anxiety scale at T1, SDQ Emotional Symptoms at T2).

We used different psychiatric screening measures across the two timepoints. The T1 DBC questionnaire was selected by focus group of parents of children with autism (see Chandler et al., 2016) at the outset of the study but we used the measure we deemed to be developmentally appropriate at each timepoint and the T2 SDQ allows comparison with a wider range of other autism studies.

#### IQ

At both T1 (intensive and extensive samples) and T2 (intensive sub‐sample only) the IQ assessment was selected according to the child's chronological age and developmental level (WPPSI‐III; WISC‐IV; MSEL; see Supplementary Materials).

#### Adaptive functioning

The Adaptive Behavior Assessment System‐II (ABAS‐II; Harrison & Oakland, 2003) was used at T2. The ABAS asks parents/carers for information across nine skill domains (Communication, Community Use, Functional Academics, Home‐living, Health/Safety, Leisure, Self‐care, Self‐direction, and Social), and domain scores are used to generate a general adaptive composite (GAC) score. Intensives completed the full ABAS (*n* = 72), whereas extensives completed the Communication domain only (*n* = 107). Multiple imputation (*n* = 179 datasets) was used to generate equivalent GAC scores for those who did not complete the full ABAS. Within the intensive sample, we found a strong association between the communication subscale and GAC scores (*r* = 0.84) and full prediction model (*R* ^2^ = 0.74) were both strong (see Supplementary Materials). To reduce the floor effect on the ABAS, scores at the floor (standard score of 40) were replaced with those generated from a prediction model that included T2 age, ABAS raw total score and ABAS ratio score (see Supplementary Materials).

### Statistical analysis

Analyses were undertaken in Stata 16 (StataCorp., 2019). Significance was set at *p* < 0.05. An attrition analysis found that T2 non‐participants (*n* = 98) had lower T1 IQs than T1 and T2 participants (*p* = 0.008) but no significant difference in T1 autism or psychiatric symptoms (see Supplementary Materials). All analyses covaried for T1 IQ and were estimated by full maximum likelihood making the analysis assumption that all other variables were missing at random (MAR).

The analysis was conducted in a stepwise fashion.

**FIGURE 1:**
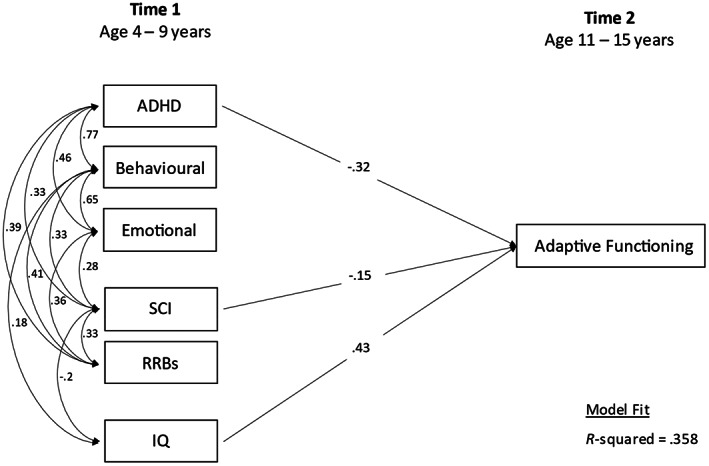
Time 1 associates of Time 2 adaptive functioning (Step 1)

*Step 1*. Examined the association between T1 characteristics and T2 adaptive function using multiple linear regression. The model allowed for covariances between all T1 symptom domains, and between these and T1 IQ (shown in Figure 1).

At each subsequent step, structural equation modeling (SEM) was used to understand the extent to which these associations were due to homotypic versus heterotypic continuity in mental health symptom domains, accounting for correlations between domains. Overall model fit was considered good when the root mean square error of approximation (RMSEA) was ≤0.05 and comparative fit index (CFI) > 0.90 (Bentler, 1990).

**FIGURE 2:**
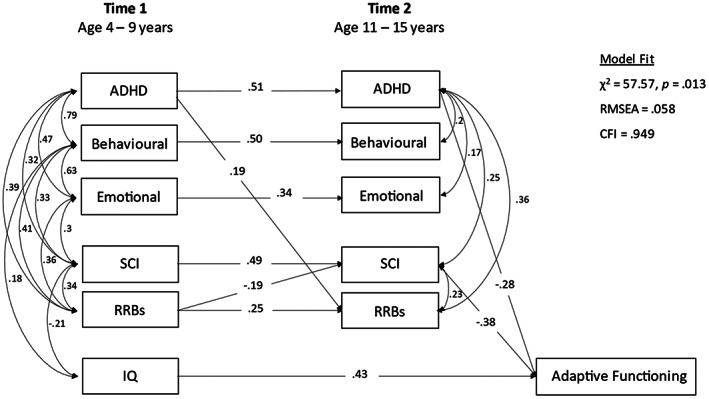
Contemporaneous associates of adaptive functioning (Step 2)

*Step 2*. Homotypic and cross‐lagged heterotypic paths from T1 to T2 psychiatric and autism symptoms, to test for within‐ and cross‐domain associations over time, accounting for correlations between domains, T2 autism and psychiatric symptoms were included as correlated outcomes with T2 adaptive functioning. This enabled us to estimate (a) within‐ and cross‐domain associations between T1 and T2 symptoms, and (b) associations between T2 symptom domains and T2 ABAS, omitting non‐significant paths to understand the “active” drivers. Direct paths between T1 symptom domains and adaptive function were excluded in this model, that is we required the effect of T1 symptoms on adaptive function to be mediated by T2 homotypic or heterotypic effects of T2 (short‐term) symptoms (shown in Figure 2). Non‐significant pathways and covariances were constrained to zero, in order of their standardized coefficients (smallest first), and with reference to model fit, with changes retained where model fit did not significantly worsen as indexed by the change in likelihood‐ratio *x* ^2^. A model that was constrained to explore only homotypic continuity, excluding paths from T1 symptoms to T2 ABAS is shown in the Supplementary Materials (Figure S2).

**FIGURE 3:**
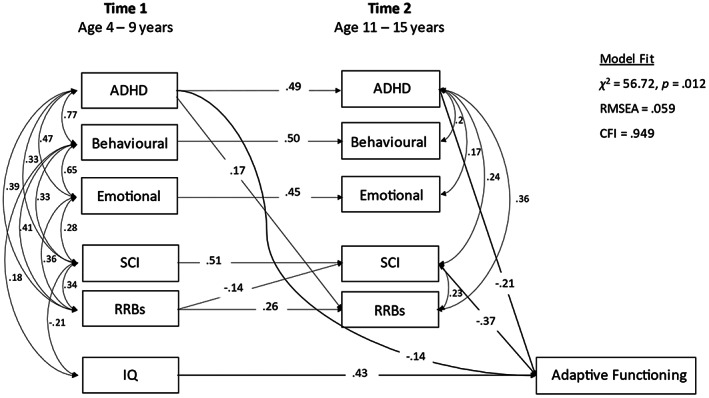
Associates of adaptive functioning: Final model (Step 3)

*Step 3*. The previous model assumed T1 symptom effects are always mediated by T2 symptoms. To examine whether this was the case, the post‐estimation command *mindices* was used to identify paths omitted from the model that would have been significant had these longer‐term effects been included, to generate the final model (shown in Figure 3). The total effects of T1 symptoms on T2 adaptive functioning were then calculated, to include direct and indirect effects, following Wright's rules of tracing (Wright, 1934).

*Step 4*. Group analyses were run for the significant paths of interest, to test for potential moderator effects of sex (males vs. females) and IQ (T1 IQ < 70 vs. IQ ≥ 70). Post‐estimation Wald tests evaluated group differences on paths of interest.

Sensitivity analyses were conducted (a) for the intensives with fully observed ABAS data (*n* = 72), and (b) repeating the imputed analysis excluding one sibling (selected at random) from three sibling pairs with non‐independent data (*n* = 176).

## RESULTS

Sample characteristics for the 179 participants included in the analysis are presented in Table 1. The mean (SD) IQ of the intensive sample at T2 was 69.0 (31.8) and for the intensive sample with full ABAS‐2 the mean GAC score at T2 was 56.3 (21.2), indicating a mean adaptive function score 12.7 (26.3) standardized score points below their measured IQ.

**TABLE 1:** Sample characteristics at Time 1 and Time 2

	Time 1	Time 2
	*N* = 179	*N* = 179
	Mean (SD), range	Mean (SD), range
Age in years	6.8 (1.2), 4.5–9.8	13.5 (1.2), 11.2–15.7
Sex: Male:Female *N* (%)	144 (80.5%):35 (19.5%)	
Ethnicity: White *N* (%)	93 (54.1%)	
IQ	75.7 (25.5), 19–129	69 (31.8), 19–129^a^
IQ < 70	59 (33.0%)	33 (46.5%)^a^
IQ ≥ 70	120 (77.0%)	38 (53.5%)^a^
SCQ total	19.5 (7.4), 1–37	16.9 (6.4), 3–31
SCQ SCI score	11.7 (5.6), 0–25	10.5 (4.6), 1–23
SCQ RRB score	7.0 (2.8), 0–12	5.5 (3.0), 0–12
DBC total	69.3 (31.1), 6–139	‐
DBC Disruptive/antisocial	21.6 (11.5), 1–48	‐
DBC Anxiety	7.6 (4.3), 0–17	‐
DBC Hyperactivity	7.4 (3.3), 0–12	‐
SDQ total	‐	16.8 (6.5), 2–34
SDQ Emotional Symptoms	‐	4.1 (2.7), 0–10
SDQ Behavioral Symptoms	‐	2.3 (1.9), 0–8
SDQ Hyperactivity Symptoms	‐	5.8 (2.7), 0–10
ABAS‐2 GAC score	‐	60.4 (18.5), 23.1–105

Abbreviations: ABAS GAC, Adaptive Behavior Assessment System General Adaptive Composite; DBC, Developmental Behavior Checklist; RRB, Restricted and Repetitive Behaviors; SCI, Social Communication and Interaction; SCQ, Social Communication Questionnaire; SDQ, Social Communication Checklist.

^a^ Available on *N* = 71.

*Step 1*. Lower adaptive functioning at T2 was associated with higher T1 ADHD (*β* = −0.32, 95% CI = −0.51, −0.13, *p* = 0.001) and T1 SCI symptoms (*β* = −0.15, 95% CI = −0.28, 0.02, *p* = 0.021) and lower T1 IQ (*β* = 0.43, 95% CI = 0.31, 0.55, *p* = 0.001) at T1 (*R* ^2^ = 0.36). Adaptive functioning was not associated with the other T1 psychiatric symptom domains (behavioral or emotional symptoms, *p* = 0.33 and *p* = 0.40), or with RRBs (*p* = 0.71) (Figure 1; see full summary of models in Table S4).

*Step 2*. Model fit was adequate (RMSEA = 0.058, CFI = 0.949; see Figure 2). Significant cross‐lagged pathways were found for T1 RRBs to T2 SCI (*β* = −0.19, 95% CI = −0.33, −0.05, *p* = 0.006), and T1 ADHD to T2 RRBs (*β* = 0.19, 95% CI = 0.04, 0.33, *p* = 0.011). At T2, ADHD was the only psychiatric domain to be associated with contemporaneous adaptive functioning, with higher ADHD scores associated with lower ABAS scores (*β* = −0.28 95% CI = −0.39, −0.17, *p* < 0.001). T2 SCI scores were also (negatively) associated with contemporaneous adaptive functioning (*β* = −0.38, 95% CI = −0.48, −0.27, *p* < 0.001).

*Step 3*. Post‐estimation analyses for omitted paths indicated that the only significant path to T2 adaptive functioning missing from Step 3 was that from T1 ADHD. This was added to the final model (Figure 3; RMSEA = 0.059, CFI = 0.949). The total effect of T1 ADHD on T2 adaptive functioning was (0.14 + 0.49 * 0.21) = 0.24, accounting for 6% of the variability in T2 adaptive functioning. The total effect of T1 SCI on T2 adaptive functioning, including via its correlation with T1 IQ was (0.51 * 0.37) + (0.21 * 0.43) = 0.28, accounting for 8% of the variability in T2 adaptive functioning.

**FIGURE 4:**
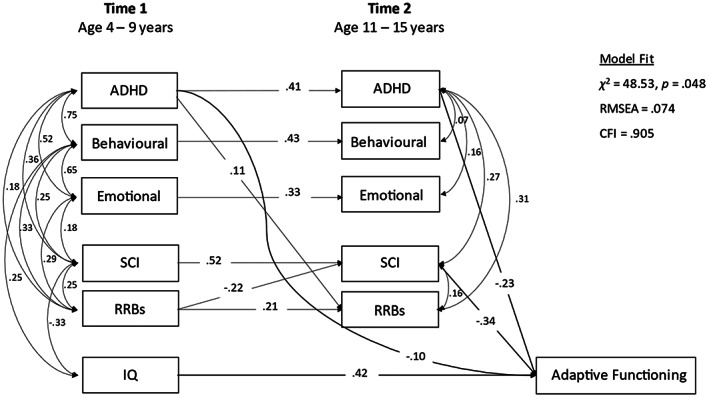
Full model (Step 3) for the intensive (*n* = 72) sample only

*Sensitivity analysis*. (a) There was no change in the findings when Steps 1–4 were repeated for cases with independent data only (*n* = 176). (b) When the model was re‐run for the intensives with fully observed ABAS data (*n* = 72), the findings remained the same in terms of early childhood and contemporaneous associates of T2 adaptive functioning (Model fit was adequate; RMSEA = 0.074, CFI = 0.905; see Figure 4). However, T1 to T2 associations for emotional problems and for RRBs no longer reached significance (*p* = 0.35 and *p* = 0.07, respectively).

*Step 4*. To test for potential moderator effects of sex and IQ, models were run which allowed associations to vary by group on the following paths of interest: T1 ADHD to T2 adaptive functioning; T1 ADHD to T2 ADHD; and T2 ADHD to T2 adaptive functioning. The overall pattern of results was similar for males versus females, and for those with T1 IQ < 70 versus IQ ≥ 70 (see Table 2). Post‐estimation Wald tests indicated a sex difference for the path from T1 ADHD to T2 ADHD (*χ* ^2^ = 4.90, *p* = 0.027). Results suggested stronger continuity in ADHD symptoms for males versus females, but this path remained significant for both males and females (*B* = 0.41, *p* < 0.001; *B* = 0.30, *p* < 0.001, respectively).

**TABLE 2:** Group analyses for sex and IQ paths of interest

Path	Coefficient (95% CI)	*p*‐value	Coefficient (95% CI)	*p*‐value	Wald test	
	Males (*n* = 144)		Females (*n* = 35)		*χ* ^2^	*p*‐value
T1 ADHD to T2 adaptive functioning	−0.77 (−1.32 to −1.01)	<0.001	−0.89 (−1.77 to 0.02)	0.045	0.139	0.709
T2 ADHD to T2 adaptive functioning	−1.48 (−2.32 to 0.64)	0.001	−1.76 (−2.97 to 0.56)	0.004	0.391	0.532
T1 ADHD to T2 ADHD	0.41 (0.31 to 0.50)	<0.001	0.30 (0.16–0.43)	<0.001	4.902	0.027

	IQ < 70 (*n* = 59)		IQ ≥ 70 (*n* = 120)		*χ* ^2^	*p*‐value
T1 ADHD to T2 adaptive functioning	−0.30 (−2.21 to 0.53)	0.032	−2.19 (−3.35 to −1.03)	<0.001	3.381	0.066
T2 ADHD to T2 adaptive functioning	−1.02 (−2.11 to 0.06)	0.052	−1.65 (−2.52 to 0.78)	<0.001	1.684	0.194
T1 ADHD to T2 ADHD	−0.38 (0.27–0.49)	<0.001	0.42 (0.31 to 0.52)	<0.001	0.977	0.323

Abbreviations: T1, Time 2; T2, Time 2.

## DISCUSSION

The primary finding from this study was that increased ADHD symptoms, both contemporaneously and in early childhood, were associated with lower adaptive functioning in autistic adolescents, above and beyond the effects of autism symptoms and IQ. The fact that the influence of ADHD on later adaptive functioning was detectable from as early as 6 years of age (range 4–9 years) is important as it is a potentially modifiable factor and early initiated treatment may effect long‐term changes in adaptive behavior trajectory. This may be especially important since whilst there is robust evidence for improvements in some aspects of social communication function from developmental‐behavioral interventions, this is not the case for adaptive functioning (see Sandbank et al., 2020).

The study extends previous cross‐sectional research, which has shown lower adaptive functioning in autistic children with higher ADHD symptoms (Sikora et al., 2012; Yerys et al., 2009, 2019). Not only was there strong within domain continuity in ADHD symptoms over time, both early childhood and adolescent ADHD symptoms were independently associated with T2 lower adaptive functioning. The associations between ADHD and adaptive functioning were not moderated by sex or IQ, although the continuity of ADHD symptoms over time was stronger for males than females, as has previously been reported in ADHD samples (Smith et al., 2017). By contrast, no effects were found for behavioral nor emotional symptoms, at either timepoint. One previous study in adults has shown a contemporaneous association between depression and anxiety symptoms and adaptive functioning (Kraper et al., 2017) but another study including children, adolescents and adults also found no association (Tillmann et al., 2019).

In common with previous cross‐sectional (Duncan & Bishop, 2015; Kanne et al., 2011; Tillmann et al., 2019) and longitudinal studies (Bal et al., 2015; Clarke et al., 2020; Szatmari et al., 2015), IQ at T1 was also positively associated with adaptive functioning at T2. In terms of autism symptoms, despite strong cross‐time continuity, higher T2 but not T1 SCI symptom severity was associated with lower adaptive functioning at T2. Tillmann et al. (2019) also found that social communication, but not repetitive and restricted behavior, symptoms were contemporaneously associated with adaptive functioning in a sample of children, adolescents, and adults. Social communication impairments may restrict an individual's opportunities for learning about and acquiring adaptive and functional skills in everyday life. However, RRB symptoms were not associated with adaptive functioning, either across time or contemporaneously. This is inconsistent with the notion that rigidity and over‐focused interests might limit the acquisition of functional skills. However, we measured RRB but not cognitive flexibility or broader executive functioning difficulties both of which have been shown to be associated with adaptive functioning (Bertollo et al., 2020; Pugliese et al., 2016); with Bertollo et al. (2020) finding that reduced flexibility was associated with lower adaptive skills in autistic youth even when ADHD symptoms were accounted for.

In part due to the lack of a measure of adaptive functioning at T1, the current analyses cannot robustly determine if the independent path (rather than via adolescent ADHD) from early childhood to later adaptive function represents measurement error or independent longer‐term influences. We also are unable to directly test the homotypic continuity of adaptive functioning across this developmental period and any potential cross‐sectional or longitudinal interaction with ADHD symptoms. Understanding the developmental interplay between ADHD symptoms and adaptive functioning in autistic youth over time will require future longitudinal cohort and intervention studies to measure these constructs at multiple timepoints to determine which cascading mechanisms may be operating and will be important to inform both potential treatment targets and optimal time periods.

Additionally, with the questionnaire measures we used we were not able to test the associations with hyperactivity/impulsivity and inattention ADHD symptom domains separately and future studies should test this. It is likely that both the core overactivity/impulsivity and inattention difficulties that characterize ADHD, and the common associated cognitive difficulties with components of the executive function system, may impair everyday functioning, acquisition and implementation of life skills and independence that make up adaptive behavior—especially in combination with having autism. Structured activity and behavioral intervention programmes that directly target and support adaptive skills such as daily living skills for autistic youth have been developed and begun to be tested for feasibility and acceptability but not yet tested for efficacy (Duncan et al., 2018, 2021). However, if the efficacy of such programmes were to be demonstrated they may be beneficial to support skill development and independence of youth with autism, in particular those with elevated levels of ADHD symptoms.

### Strengths and limitations

The study has several strengths. We studied a community sample of all young children who received an autism diagnosis from local services; we purposefully over‐sampled girls to allow us to investigate sex differences; and we included a wide range of IQ and covaried for the effects of IQ that was also related to sample attrition. Therefore, our findings may be generalisable to the wider population of autistic children. We measured autism symptoms and psychiatric symptoms at both timepoints, which allowed us to examine developmental and contemporaneous effects independently. Our statistical analysis took into account covariances between autism, psychiatric symptom domains and IQ, and provides a parsimonious test of the unique associations between the constructs of interest.

However, the study also has some limitations. Our stratified study design meant we imputed ABAS GAC for a defined sub‐sample who only completed the Communication domain of the ABAS, although the association between imputed and observed and the full prediction model were both strong. We measured adaptive behavior at T2 and not T1, and IQ at T2 in the intensive sub‐sample only, so were only able to calculate the level of IQ‐adaptive function discrepancy at T2 and not whether this changed across development in our sample. We used different psychiatric screening measures across the two timepoints (DBC at T1, SDQ at T2). However, we deemed them the most developmentally appropriate at each timepoint given the wide range of IQ in the sample. The correlation between the measures in non‐autistic youth with intellectual disability is strong (Swift et al., 2021). Both the DBC and SDQ subscales measure aspects of all the components of ADHD, including inattention, overactivity and impulsivity and in all three psychiatric symptom domains including ADHD homotypic continuity between T1 and T2 was strong (see Figure S2). Our study used parent report of all the key constructs, except IQ, so shared method variance may account for some of the associations found and future studies should use other methods such as investigator‐based psychiatric interviews and observational measures. A recent study found that positive family functioning was associated with later adaptive socialization abilities from early to mid‐childhood (Szatmari et al., 2021). Environmental factors as well as child factors may influence the development of adaptive skills over development and should be considered in future studies (Hollocks & Simonoff, 2021).

### Clinical implications

We demonstrate that ADHD symptoms—both earlier in development and contemporaneously—are associated with adaptive functioning outcomes in autistic adolescents. This is important because such symptoms may be improved by intervention, with the potential for knock‐on beneficial effects on adaptive functioning. An evidence‐base exists both for pharmacological treatments and psychological interventions and environmental modifications that can ameliorate ADHD symptoms in non‐autistic youth (Coghill et al., 2021; National Institute for Health and Care Excellence, 2018). However, their effectiveness has only recently been systematically assessed in autistic youth. Whilst behavioral parenting interventions suggest some effect in reducing ADHD symptoms (Tarver et al., 2019), in common with the wider ADHD literature (Sonuga‐Barke et al., 2013) few studies to date have used blinded, objective outcome measures. There is evidence supporting the use of methylphenidate, atomoxetine and guanfacine to reduce ADHD symptoms in autistic youth, although the effect sizes for methylphenidate and atomoxetine achieved are lower and the rate of adverse events higher for methylphenidate than those seen in non‐autistic children (Rodrigues et al., 2020).

### Conclusions

Co‐occurring ADHD should be routinely assessed in young autistic children and when elevated levels are identified evidence‐based treatments initiated. Such treatment may have beneficial effects on broader later adaptive functioning outcomes.

## AUTHOR CONTRIBUTIONS

Drs Tony Charman, Emily Simonoff, Andrew Pickles, and Gillian Baird obtained funding, conceived and designed the study, and were involved in the interpretation of data, and drafting and critical revision of the manuscript for important intellectual content. Dr Susie Chandler had full access to all of the data in the study and takes responsibility for the integrity of the data and the accuracy of the data analysis, and was involved in the interpretation of data, and drafting and critical revision of the manuscript for important intellectual content. Drs Virginia Carter Leno, Philippa White, Isabel Yorke, and Matthew J. Hollocks were involved in the interpretation of data, and drafting and critical revision of the manuscript for important intellectual content. All authors approved the final manuscript as submitted and agree to be accountable for all aspects of the work.

## FUNDING INFORMATION

The original QUEST Study was funded by the Clothworkers' Foundation, brokered by Research Autism (R011217 Autism M10 2011/12). The study was part of a wider programme, Improving Autism Mental Health (IAMHealth) funded by the National Institute for Health Research (NIHR) (RP‐PG‐1211‐20016). Dr Virginia Carter Leno is supported by a Sir Henry Wellcome Postdoctoral Fellowship (213608/Z/18/Z). Drs Emily Simonoff and Andrew Pickles receive NIHR Senior Investigator Awards (NF‐SI‐0514‐10073 and NF‐SI‐0617‐10120). The other authors received no external funding. The study was partially supported through the NIHR Maudsley Biomedical Research Centre at the South London and Maudsley NHS Foundation Trust in partnership with King's College London. The views expressed are those of the authors and not necessarily those of the NHS, or the NIHR.

## CONFLICTS OF INTEREST

Dr. Tony Charman has served as a paid consultant to F. Hoffmann‐La Roche Ltd. and Servier; and has received royalties from Sage Publications and Guilford Publications. Dr Andrew Pickles receives royalties from WPS, OUP and ICP publishing. The other authors have no relevant conflicts to disclose.

## Supporting information


**Appendix S1** Supporting information

## Data Availability

The data that support the findings of this study are available from the corresponding author upon reasonable request.

## Footnotes

^1^
We use “autism” to refer to Autism Spectrum Disorder (ASD; DSM‐5 (American Psychiatric Association, 2013)) and “autistic individuals” as this is the preferred term of some in the autism community (Kenny et al., 2016).
