# Supplementary material for: Pathways to adaptive functioning in autism from early childhood to adolescence
Source: Autism Res. Author manuscript; Available in PMC 2023 Jan 3. (PMC9796413; doi:10.1002/aur.2785)
Supplement: Supplementary Materials [file EMS158861-supplement-Supplementary_Materials.docx]

**Supplementary Materials**

**Table S1.** ADOS and ADI-R scores for intensive subsample (where available)

|  | Mean (SD), range |
| --- | --- |
| ADOS^a^ |  |
| SA score | 11.1 (4.8), 0 – 20 |
| RRB score | 2.3 (2.4), 0 – 8 |
| Total score | 13.4 (6.5), 1 – 26 |
| Module 1 N (%)  Module 2 N (%)  Module 3 N (%) | 16 (22.5%)  7 (9.9%)  47 (67.6%) |
| Comparison score | 6.6 (2.6), 1 – 10 |
| Classification non-spectrum N (%)  Classification autism spectrum N (%)  Classification autism N (%) | 11 (15.5%)  9 (12.7%)  51 (71.8%) |
| ADI-R^b^ |  |
| RSI score  Communication score  RRB score | 18 (5.3), 4 – 27  13.7 (4.5), 3 – 23  4.4 (2.4), 0 – 9 |
| Classification non-spectrum N (%)  Classification autism spectrum N (%)  Classification autism N (%) | 2 (3.9%)  8 (15.7%)  19.6 (80.4%) |

Note. a = Available on *N* = 71; b = Available on *N* = 51. ADOS = Autism Diagnostic Observation Schedule; SA = Social Affect; RRB = Restricted and Repetitive Behaviours; ADI-R = Autism Diagnostic Interview-Revised; RSI = Reciprocal Social Interaction.

*Co-occurring psychiatric symptoms.* At Time 1, the 96-item parent/carer version of the Developmental Behavior Checklist version (DBC; Einfeld & Tonge, 2002) was used covering a broad range of behaviours, each rated as 0 (‘not true as far as I know’), 1 (‘somewhat or sometimes true’) or 2 (‘very or often true’). The DBC has five empirically derived subscales (Disruptive/Antisocial; Self-absorbed; Communication Disturbance; Anxiety; and Social-relating) and four additional scales that can be used to identify specific psychiatric syndromes (Autism Screening Algorithm; Depression scale; Hyperactivity scale; Anxious Behaviour Rating scale).

At Time 2, the parent/carer report Strengths and Difficulties Questionnaire (SDQ; Goodman, Ford, Simmons, Gatward & Meltzer, 2000) was used. The SDQ comprises 25 items, and the subscales: Emotional Symptoms; Conduct Problems; Hyperactivity-inattention; Peer Relationships; and Pro-social Behaviours. Each subscale comprises 5 items scored ‘Not True’, ‘Somewhat True’ and ‘Certainly True’ with some items on each subscale reverse scored (0/1/2).

**IQ assessments at Time 1 and Time 2**

At Time 1, the IQ assessment was selected according to the child’s chronological age and developmental level: 48 children completed the Wechsler Intelligence Scale for Children-IV (WISC-IV; Wechsler, 2004) 101 the Wechsler Preschool and Primary Scale of Intelligence-III (WPPSI-III; Wechsler, 2002), and 24 the Mullen Scales of Early Learning (MSEL; Mullen, 1995). When the WPPSI and MSEL were used out of age range, age-equivalents were calculated, and a ratio IQ derived [ratio IQ = (age-equivalent/chronological age) x 100]. Those with a MSEL ratio IQ < 20 were assigned an IQ of 19 to reflect their very low ability. Six children did not have an IQ from a standardised test (either because they could not access the test, or because it was not possible to see them). Parental estimates of functional age were also used to generate a parent-estimated developmental quotient [DQ = (estimated functional age/chronological age) x 100] at Time 1. As with the low ratio IQs from the MSEL, those with a parent-estimated DQ<20 were assigned a DQ of 19 to reflect their low ability. For those with both a test IQ and parent-estimated DQ, the two scores were strongly correlated (r (151) = .72, *p*<.0001). 58% of parent-estimated DQs were within 1 standard deviation of the test IQ; 91% were within 2 standard deviations. Therefore, for the 6 children lacking a test IQ at Time 1, the parent-derived DQ was used instead.

At Time 2, 48 intensives completed the Wechsler Abbreviated Scale of Intelligence-II (WASI-II; Wechsler, 2011), 10 the WPPSI-IV (Wechsler, 2012), and 13 the MSEL. Where assessments were used out of age range, ratio IQs were again calculated as described above.

**Reducing the ABAS floor effect**

Sixteen intensives and two extensives (10% of this sample) had Adaptive Behavior Assessment System (ABAS) general adaptive composite (GAC) standard scores of 40, the lowest possible score. To reduce this floor effect, we used predictive modelling within the intensive subsample to fit ABAS GAC scores on the basis of Time 2 age, ABAS raw total, and ABAS ratio score (ratio score = (ABAS raw total/age)). The model included log and exponential functions to ensure fitted scores fell within a valid range (i.e., no negative scores), with the resulting model predicting 95% of the variance in ABAS GAC standard scores (see Figure S1). For the 16 intensives with ABAS GAC scores of 40, these were therefore replaced with the fitted ABAS scores. It was not possible to run the model for the extensives as they lacked an ABAS raw total.

**Imputation of ABAS GAC for participants completing only the Communication domain.**

Intensives completed the full ABAS, whereas extensives completed the Communication items only. Parent/carers of minimally verbal children completed the Communication items for 0-5 years, rather than items appropriate for their chronological age. For the extensives, scores for the other 8 skill domains were calculated for the full sample using multiple imputation (100 imputations), with Time 2 ABAS Communication scaled score, Time 2 age, Time 2 school type, along with stratification variables from Time 1 (IQ, borough, sex and SCQ score) included in the model. Imputations were only calculated where all variables included in the model were present. Imputed domain scores, based on the aggregated test statistic across the imputed datasets, were then used to generate GAC scores for those who did not complete the full ABAS. This yielded ABAS GAC scores for 179 participants, of which 72 were based on observed domain scores and 107 were based on imputed domain scores. For the 72 with fully observed data, ABAS GAC and Communication scaled scores were highly correlated, *r* (72) = .84 (*p*<.0001); and the prediction of ABAS GAC from the variables included in the imputation models was strong (*R*^2^ = .74, F (7, 63) = 26.23, *p* <.0001).

**Figure S1**. Fitting ABAS GAC standard scores on the basis of age, ABAS raw total and ABAS ratio score

*R*^2^ = 0.95

RMSE = 0.06

Note. ABAS = Adaptive Behaviour Assessment System; GAC = General Adaptive Composite; RMSE = root mean square error

**Table S2.** Descriptive statistics for the 16 intensives with ABAS GAC scores at the floor

|  | Mean (SD), range |
| --- | --- |
| Fitted ABAS GAC score | 30.1 (5.3), 23.1 – 40.0 |
| Time 2 age in years | 13.5 (1.2), 11.4 – 15.6 |
| ABAS raw total | 119.3 (65.8), 25 – 237 |
| ABAS ratio score | 8.8 (4.8), 1.9 – 16.7 |
| Time 1 IQ | 35.4 (19.6), 19 – 86 |

Note. ABAS = Adaptive Behavior Assessment System; GAC = General Adaptive Composite

**Table S3**. Sample characteristics for participants versus non-participants

|  | Participants | Non-participants |
| --- | --- | --- |
| Time 1 characteristics | *N* = 179  *Mean (SD), range* | *N* = 98^a^  *Mean (SD), range* |
| IQ | 75.7 (1.9), 19 – 129* | 66.7 (2.7), 19-119* |
| SCQ total  SCQ SCI score  SCQ RRB score | 19.5 (7.4), 1 – 37  11.7 (5.6), 0 – 25  7.0 (2.8), 0 – 12 | 21.0 (7.4), 5 – 36  12.8 (5.8), 1 – 24  7.1 (3.1), 0 – 12 |
| DBC total  DBC Disruptive/antisocial  DBC Anxiety  DBC Hyperactivity | 69.3 (31.1), 6 – 139  21.6 (11.5), 1 – 48  7.6 (4.3), 0 – 17  7.5 (3.4), 0 – 12 | 74.7 (26.4), 18 – 141  22.6 (9.4), 6 – 43  8. 23 (3.9), 0 – 18  8.1 (2.8), 0 – 12 |

Note. a = Non-participants comprised Time 1 participants who did not participate at Time 2 (n = 66), individuals who participated at both timepoints but did not complete the ABAS (n = 14), minimally verbal extensives lacking an ABAS Communication scaled score required for the imputation (n = 13), extensives missing any other variable required for the ABAS imputation (n = 5).

SCQ = Social Communication Questionnaire; SCI = Social Communication and Interaction; RRB = Restricted and Repetitive Behaviours; DBC = Developmental Behavior Checklist

*independent t-test, *p* <.05

**Table S4.** Summary of model fit statistics for regression (Step 1) and SEM models (Steps 2 to 5)

|  |  | **χ^2^ ms** | **χ^2^ sig** | | **Δχ^2^** | | **Δχ^2^ Sig** | | **DF** | | **CFI** | | **RMSEA** | |
| --- | --- | --- | --- | --- | --- | --- | --- | --- | --- | --- | --- | --- | --- | --- |
| *Step 1: Testing longitudinal associations with adaptive functioning* | | | | | | | | | | | | | | |
| 1.1 | Associations between T1 autism and psychiatric symptoms and T2 adaptive functioning. T1 IQ included. Co-variances allowed. Saturated model | 0.00^a^ | - | | ^b^ | | - | | 35 | | 1.000 | | 0.000 | |
| 1.2 | Constrain path T1 RRB -> adaptive functioning | 0.14 | .713 | | - | | - | | 34 | | 1.000 | | 0.000 | |
| 1.3 | Constrain path T1 Emotional -> adaptive functioning | 0.94 | .625 | | 0.81 | | .370 | | 33 | | 1.000 | | 0.000 | |
| 1.4 | Constrain path T1 Behavioural -> adaptive functioning | 1.29 | .732 | | 0.35 | | .557 | | 32 | | 1.000 | | 0.000 | |
| 1.5 | Constrain co-variance between T1 RRB and IQ | 1.29 | .862 | | 0.01 | | .929 | | 31 | | 1.000 | | 0.000 | |
| 1.6 | Constrain co-variance between T1 ADHD and IQ | 2.24 | .816 | | 0.94 | | .332 | | 30 | | 1.000 | | 0.000 | |
| 1.7 | Constrain co-variance between T1 Emotional and IQ | 6.08 | .414 | | 3.84 | | .0500 | | 29 | | 0.999 | | 0.009 | |
| Step 1 final model R^2^  = .358 | | | | | | | | | | | | | | |
| *Step 2: Testing whether associations between T1 symptoms and T2 adaptive functioning are due to their longitudinal continuity and contemporaneous effect on adaptive function* | | | | | | | | | | | | | | |
| 2.1 | Add T2 autism and psychiatric symptoms | 65.96 | | .001 | | ^b^ | | - | | 57 | | 0.922 | | 0.075 |
| 2.2 | Constrain path T2 Behavioural -> adaptive functioning | 66.81 | | .001 | | 0.85 | | .357 | | 56 | | 0.922 | | 0.074 |
| 2.3 | Constrain path T2 Emotional -> adaptive functioning | 66.91 | | .001 | | 0.10 | | .754 | | 55 | | 0.925 | | 0.072 |
| 2.4 | Constrain path T2 RRB -> adaptive functioning | 69.28 | | .001 | | 2.37 | | .123 | | 54 | | 0.921 | | 0.072 |
| 2.5 | Constrain co-variance between T2 Behavioural and T2 Emotional | 69.34 | | .001 | | 0.06 | | .813 | | 53 | | 0.924 | | 0.070 |
| 2.6 | Constrain co-variance between T2 Emotional and T2 SCI | 69.45 | | .001 | | 0.11 | | .739 | | 52 | | 0.926 | | 0.068 |
| 2.7 | Constrain co-variance between T2 Behavioural and T2 SCI | 71.70 | | .0001 | | 2.25 | | .134 | | 51 | | 0.923 | | 0.069 |
| 2.8 | Constrain co-variance between T2 Emotional and T2 RRB | 74.30 | | .001 | | 2.60 | | .107 | | 50 | | 0.919 | | 0.070 |
| 2.9 | Constrain co-variance between T2 Behavioural and T2 RRB | 76.33 | | .001 | | 2.02 | | .155 | | 49 | | 0.916 | | 0.070 |
| *Step 3: Testing for cross-domain associations from T1 to T2* | | | | | | | | | | | | | | |
| 3.11 | Add cross-lagged paths from T1 autism and psychiatric symptoms | 45.97 | | .001 | | ^b^ | | - | | 69 | | .941 | | 0.082 |
| 3.12 | Constrain path T1 RRB -> T2 Behavioural | 45.97 | | .002 | | .01 | | .941 | | 68 | | .943 | | 0.078 |
| 3.13 | Constrain path T1 Emotional -> T2 ADHD | 45.98 | | .003 | | 0.01 | | .940 | | 67 | | .946 | | 0.075 |
| 3.14 | Constrain path T1 SCI -> T2 ADHD | 45.99 | | .004 | | 0.02 | | .899 | | 66 | | .948 | | 0.072 |
| 3.15 | Constrain path T1 Emotional -> T2 RRB | 46.02 | | .006 | | 0.03 | | .862 | | 65 | | .950 | | 0.069 |
| 3.16 | Constrain path T1 Behavioural -> T2 SCI | 46.08 | | .009 | | 0.05 | | .819 | | 64 | | .953 | | 0.066 |
| 3.17 | Constrain path T1 ADHD -> T2 Behavioural | 46.17 | | .012 | | 0.10 | | .754 | | 63 | | .955 | | 0.063 |
| 3.18 | Constrain path T1 SCI -> T2 RRB | 46.73 | | .0146 | | 0.55 | | .457 | | 62 | | .956 | | 0.061 |
| 3.19 | Constrain path T1 Emotional -> T2 Behavioural | 47.24 | | .018 | | 0.52 | | .472 | | 61 | | .957 | | 0.060 |
| 3.20 | Constrain path T1 Behavioural -> T2 ADHD | 47.65 | | .022 | | 0.41 | | .524 | | 60 | | .958 | | 0.058 |
| 3.21 | Constrain path T1 ADHD -> T2 SCI | 48.69 | | .023 | | 1.04 | | .307 | | 59 | | .958 | | 0.057 |
| 3.22 | Constrain path T1 SCI -> T2 Behavioural | 50.58 | | .020 | | 1.89 | | .170 | | 58 | | .956 | | 0.057 |
| 3.23 | Constrain path T1 Behavioural -> T2 RRB | 50.84 | | .024 | | 0.26 | | .610 | | 57 | | .958 | | 0.055 |
| 3.24 | Constrain path T1 ADHD -> T2 Emotional | 51.97 | | .025 | | 1.13 | | .288 | | 56 | | .962 | | 0.055 |
| 3.25 | Constrain path T1 RRB -> T2 ADHD | 54.41 | | .019 | | 2.45 | | .117 | | 55 | | .954 | | 0.056 |
| 3.26 | Constrain path T1 SCI -> T2 Emotional | 57.57 | | .013 | | 3.15 | | .076 | | 54 | | .949 | | 0.058 |
| *Step 4: Adding significant omitted path* | | | | | | | | | | | | | | |
| 4.1 | Add path T1 ADHD -> adaptive functioning | 56.72 | | .012 | | 0.85 | | .356 | | 55 | | .949 | | 0.059 |
| *Step 5: Testing for moderator effects of sex and IQ* | | | | | | | | | | | | | | |
| 5.1 | Group analysis run for sex on path T1 ADHD -> T2 ADHD | 146.03 | | .096 | | ^b^ | | - | | 56 | | .948 | | 0.044 |
|  | Group analysis run for sex on path T1 ADHD -> adaptive functioning | 57.80 | | .034 | | ^b^ | | - | | 56 | | .764 | | 0.071 |
|  | Group analysis run for IQ on path T2 ADHD -> adaptive functioning | 397.52 | | <.001 | | ^b^ | | - | | 56 | | .293 | | 0.157 |
|  | Group analysis run for IQ on path T1 ADHD -> t2 ADHD | 398.48 | | <.001 | | ^b^ | | - | | 56 | | .291 | | 0.158 |
|  | Group analysis run for IQ on path T1 ADHD to adaptive functioning | 277.20 | | <.001 | | ^b^ | | - | | 56 | | .121 | | 0.258 |

^a^No *X*^2^ provided for model vs saturated model, as models equivalent.

^b^Not possible to test Δχ^2^ as models not nested.

χ^2^ ms = chi-square test of model vs saturated; χ^2^ sig = chi-square significance; Δχ^2 =^ change in chi-square from previous chi-square; Δχ^2^ Sig = chi-square change significance; DF = degrees of freedom; CFI = comparative fit index; RMSEA: root mean square of approximation; SCI = Social Communication and Interaction; RRB = Restricted and Repetitive Behaviour; T1 = Time 1; T2 = Time 2

**Figure S2**. Homotypic continuity of symptoms from T1 to T2 and contemporaneous associates of adaptive function


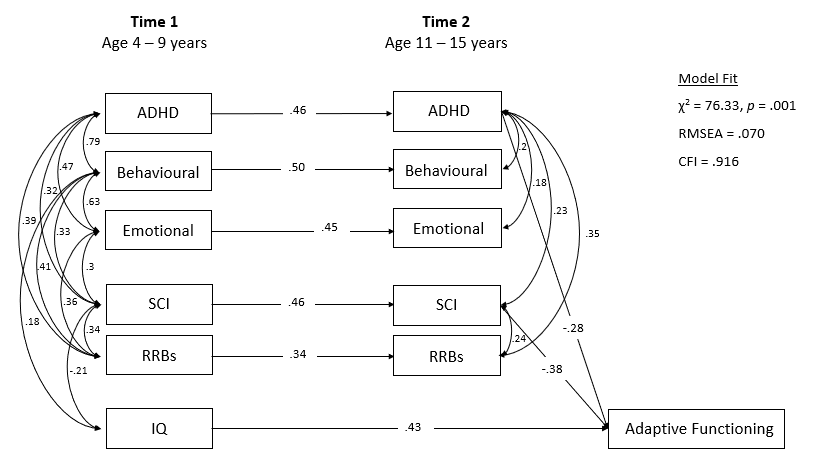


Note: Only significant paths (with their standardised coefficients) and covariances are shown; all *p*-values <.05

SCI = Social Communication and Interaction; RRB = Restricted and Repetitive Behaviour; T1 = Time 1; T2 = Time 2; RMSEA = root mean square error of approximation; CFI = comparative fit index

For a model that was constrained to explore only homotypic continuity, excluding paths from T1 symptoms to T2 ABAS fit was adequate (RMSEA = .075, CFI = .916). All symptoms showed within-domain continuity from T1 to T2: ADHD (*β* = 0.46, 95*%* CI = 0.35, 0.57, *p* < .001), emotional (*β* = 0.45, 95*%* CI = 0.33, 0.57, *p* < .001), and behavioural symptoms (*β* = 0.50, 95*%* CI = 0.39, 0.61, *p* < .001); SCI (*β* = 0.46, 95*%* CI = 0.35, 0.57, *p* < .001) and RRBs (*β* = 0.34, 95*%* CI = 0.21, 0.47, *p* < .001), indicating persistence of symptoms over a 7 year time period.

**References**

Mullen, E. (1995). *Mullen Scales of Early Learning*. Circle Pines, MN: AGS.

Wechsler, D. (2002). *Wechsler Preschool and Primary Scale of InteIligence - Third Edition*. San Antonio, TX: The Psychological Corporation.

Wechsler, D. (2004). *Wechsler Intelligence Scale for Children - Fourth Edition (UK)*. London: Harcourt Assessment.

Wechsler, D. (2011). *Wechsler Abbreviated Scale of Intelligence*. San Antonio, TX: NCS Pearson.

Wechsler, D. (2012). *Wechsler Preschool and Primary Scale of Intelligence - Fourth Edition*. San Antonio, TX: The Psychological Corporation.
